# Aging influences steroid hormone release by mink ovaries and their response to leptin and IGF-I

**DOI:** 10.1242/bio.016436

**Published:** 2016-01-21

**Authors:** Alexander V. Sirotkin, Dušan Mertin, Karin Süvegová, Abdel Halim Harrath, Jan Kotwica

**Affiliations:** 1 Department of Zoology and Anthropology, Constantine the Philosopher University, Nitra 949 74, Slovakia; 2 Department of Genetics and Reproduction, Research Institute of Animal Production, Lužianky 949 59, Slovakia; 3 Department of Zoology, College of Science, King Saud University, P.O. Box 2455, Riyadh 11451, Saudi Arabia; 4 Institute of Animal Reproduction and Food Research, Polish Academy of Sciences, Olsztyn 10-747, Poland

**Keywords:** Aging, Ovary, Mink, Leptin, IGF-I, Progesterone, Estradiol

## Abstract

The aim of our study was to understand whether ovarian steroid hormones, and their response to the metabolic hormones leptin and IGF-I leptin, could be involved in the control of mink reproductive aging via changes in basal release of ovarian progesterone and estradiol. For this purpose, we compared the release of progesterone and estradiol by ovarian fragments isolated from young (yearlings) and old (3-5 years of age) minks cultured with and without leptin and IGF-I (0, 1, 10 or 100 ng/ml). We observed that isolated ovaries of older animals produced less progesterone but not less estradiol than the ovaries of young animals. Leptin addition stimulated estradiol release by the ovarian tissue of young animals but inhibited it in older females. Leptin did not influence progesterone output by the ovaries of either young or older animals. IGF-I inhibited estradiol output in young but not old animals, whereas progesterone release was inhibited by IGF-I irrespective of the animal age. Our observations demonstrate the involvement of both leptin and IGF-I in the control of mink ovarian steroid hormones release. Furthermore, our findings suggest that reproductive aging in minks can be due to (a) reduction in basal progesterone release and (b) alterations in the response of estradiol but not of progesterone to leptin and IGF-I.

## Abstract (precis)

**Summary:** Both leptin and IGF-I are involved in the control of mink ovarian steroid hormone release. Reproductive aging can be due to (a) reduction in basal progesterone release and (b) alterations in the response of estradiol but not of progesterone to leptin and IGF-I.

## INTRODUCTION

The age-dependent reduction in animal and human fecundity (reproductive aging) can be due to the reduced release of ovarian steroid hormones in females – mainly estrogen, estradiol and the progestogen, progesterone. These hormones promote ovarian follicullogenesis, oogenesis, ovulation and gravidity (Broekmans et al., 2009; Dias et al., 2014; Finch, 2014; Hale et al., 2014). The age-dependent decline in the plasma level of other hormones – growth hormone and insulin-like growth factor I (IGF-I) – which can impair puberty and fecundity in mice and can be involved in the promotion of reproductive aging have been reported (Traub and Santoro, 2010; Junnila et al., 2013; Bao et al., 2014). IGF-I is considered a metabolic hormone controlling ovarian steroidogenesis and other ovarian functions (Sirotkin et al., 2005, 2014; Karamouti et al., 2008; Silva et al., 2009; Sirotkin, 2014) and suppressing reproduction in inadequate metabolic conditions (Chagas et al., 2007; Lucy, 2008). Leptin is another metabolic hormone regulating reproduction. The importance of leptin in the control of ovarian functions (including direct effect on ovarian cell steroidogenesis) has been well documented (Sirotkin et al., 2005, 2014; Karamouti et al., 2008; Sirotkin and Meszarosová, 2010; Chou and Mantzoros, 2014; Sirotkin, 2010, 2014). The age-dependent changes in human plasma leptin levels indicate leptin's involvement in the control of human puberty (Moshtaghi-Kashanian and Razavi, 2009; El-Eshmawy et al., 2010). Nevertheless, the direct evidence for the involvement of these metabolic hormones in the control of reproductive aging (age-dependent changes in leptin and IGF-I effects on reproduction) is lacking. In addition, the involvement of metabolic hormones leptin and IGF-I in the control of reproduction has been shown only in a small number of species (rodents, cows, pigs, chicken and humans; Sirotkin, 2014; Sirotkin et al., 2014). The role of these hormones in the control of ovarian functions in other species remains unknown.

Mink (*Mustela vison*) is a good model for the study of the endocrine control of reproduction and aging because these animals have numerous reproductive problems and age-dependent changes in fecundity. The farm breeding of these fur animals is important from an economical viewpoint, but the hormonal control of mink reproduction is insufficiently studied (Sundqvist et al., 1989; Amstislavsky et al., 2008). The release and involvement of several hormones (including progesterone, estradiol and IGF-I) in the control of mink reproduction have been reported (Sundqvist et al., 1989; Tauson et al., 2000; Tauson, 2001). Nevertheless, the role of leptin and IGF-I in the control of mink ovarian hormones release has not been studied. The endocrine mechanisms of mink reproductive aging remain completely unknown.

The aim of our study was to understand whether ovarian steroid hormones and their response to the metabolic hormones leptin and IGF-I could be involved in the control of mink reproductive aging, i.e. whether aging can be associated with changes in the basal release of ovarian progesterone and estradiol and in their response to IGF-I and leptin. For this purpose, we compared the release of progesterone and estradiol by ovarian fragments isolated from young and old minks and cultured with and without leptin and IGF-I.

## RESULTS AND DISCUSSION

**Fig. 1.**
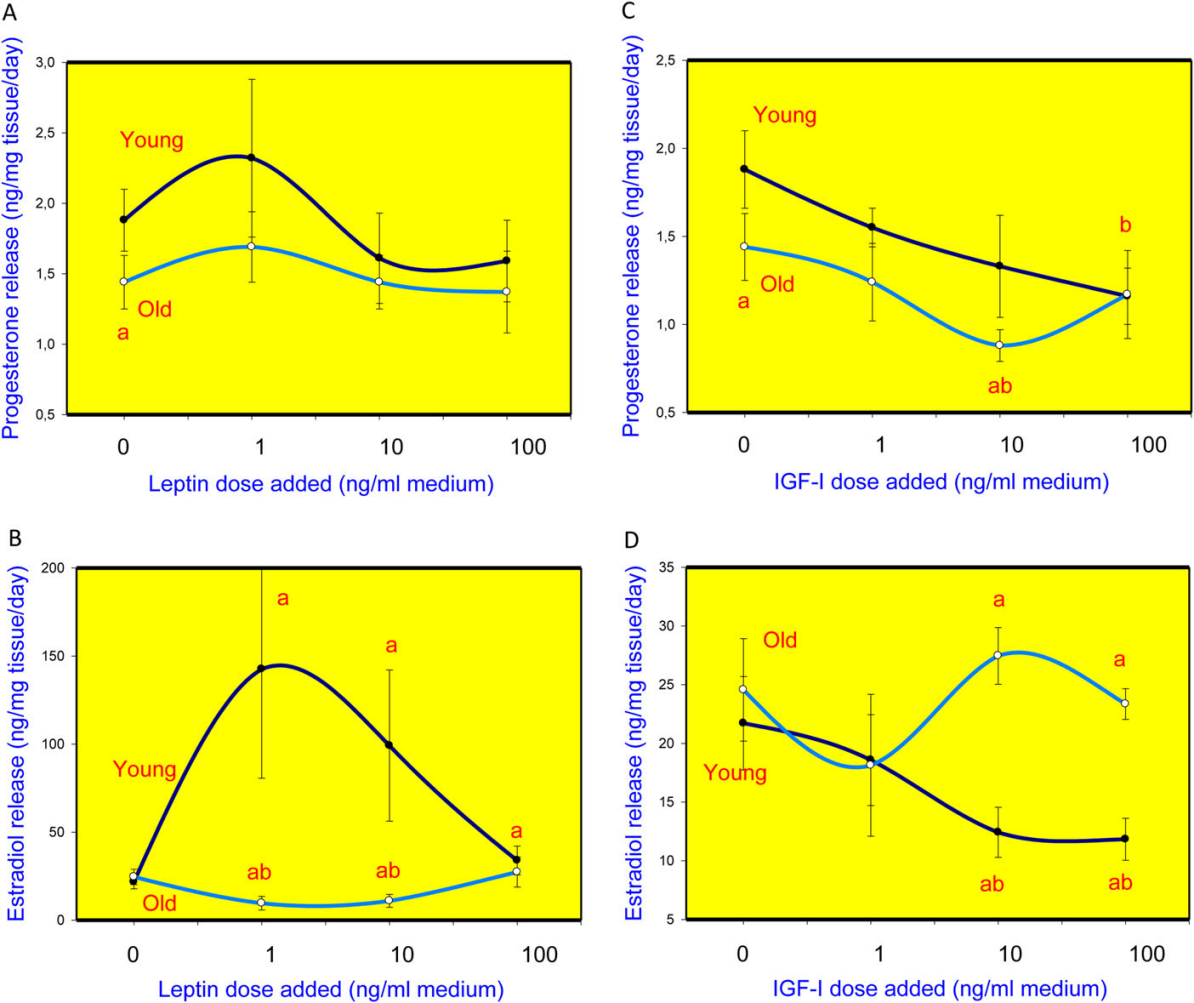
**The influence** **of leptin and IGF-I on progesterone and estradiol release.** Release of progesterone (A,C) and estradiol (B,D) by cultured fragments of ovaries isolated from young (1 year of age) and old (3-5 years of age) minks treated with leptin (A,B) and IGF-I (C,D). The data shown are the means±s.e.m. of values obtained in two separate experiments performed on separate days with separate groups of ovaries, each obtained from 10-15 animals. Each experimental group was represented by six culture wells. ‘a’ indicates a significant (*P*<0.05) difference between corresponding groups of young and old animals; ‘b’ indicates significant (*P*<0.05) differences between hormone (1, 10 or 100 ng/ml) treated and control (0 ng/ml) ovarian fragments, ‘ab’ indicates a significant (*P*<0.05) effect of both factors (age and hormone addition) by two-way ANOVA and Wilcoxon–Mann–Whitney multiple range test.

Cultured ovarian fragments produced and released progesterone and estradiol, with this process dependant on the age of animals and on the addition of leptin and IGF-I (Fig. 1). The ovarian tissue isolated from older animals and cultured without leptin or IGF-I (0 ng/ml) released significantly less progesterone than the tissue isolated from the ovaries of young minks (Fig. 1A,C). The ovaries of young and those of old animals released similar amounts of estradiol (Fig. 1B,D).

Leptin administration did not substantially affect progesterone release by the ovaries isolated from either young or older minks (Fig. 1A). Leptin addition at doses of 1 or 10 ng/ml (but not at the highest dose, 100 ng/ml) dramatically stimulated estradiol release by the ovarian tissue of young animals but inhibited it in the ovarian tissue of older females (Fig. 1B). IGF-I inhibited progesterone release by the ovarian tissue of both young (at dose 100 ng/ml) and older minks (at doses 10 and 100 ng/ml) (Fig. 1C). Furthermore, IGF-I (at 10 or 100 ng/ml) suppressed estradiol output by the ovaries of young animals but not that by the ovaries of older animals (Fig. 1D).

Our observations confirm the previous reports (Sundqvist et al., 1989; Tauson et al., 2000) of the release of steroid hormones progesterone and estradiol by mink ovaries. These hormones are considered important regulators of reproduction in this (Tauson et al., 2000; Tauson, 2001) and other (Sirotkin, 2014) species. The age-dependent changes in the release of progesterone and estradiol and their response to leptin and IGF-I provide the first evidence that these hormones are involved in the control of mink reproductive aging.

The leptin-induced changes in estradiol release demonstrate the involvement of leptin in the control of reproductive processes in minks. These data are in line with the previous reports (Sirotkin, 2010) on the involvement of leptin in the control of ovarian steroidogenesis in other species. We observed the opposite action of leptin on this steroid in young and older animals (up-regulation of estradiol output in young females and its down-regulation in older animals). The fine mechanisms of such quantitative age-dependent changes in response to leptin remain unknown, but they indicate the involvement of leptin, not only in the control of ovarian steroidogenesis but also in ovarian aging. Both leptin and estradiol are considered promoters of ovarian function and fecundity (Sirotkin, 2010, 2014). Therefore, it is possible, that the reproductive aging in minks, which is associated with a decrease in ovarian steroidogenesis and pup production (Sundqvist et al., 1989; Tauson et al., 2000; Tauson, 2001), can be induced by qualitative changes in ovarian response to leptin. In young animals, the positive, self-stimulating feedback relationships within the leptin-estradiol axis can occur, whereas aging can be associated with development of negative feedback interrelationships between leptin and estradiol.

Our observations demonstrate the involvement of IGF-I in the control of mink ovarian steroidogenesis, whereas both down-regulation (estradiol) and up-regulation (progesterone) of ovarian steroids by IGF-I is possible. These observations are in line with previous publications (Silva et al., 2009; Sirotkin, 2014; Sirotkin et al., 2014) concerning involvement of IGF-I in control of ovarian steroidogenesis in other species. Furthermore, the opposite action of IGF-I on estradiol release by the ovarian tissue of young and older minks observed in our experiments suggest that ovarian aging can be induced by changes in the IGF-I-estradiol axis: in older animals IGF-I became not an inhibitor but a stimulator of estradiol release. The causes, mechanisms and physiological significance of such changes remain to be studied, but it is possible that qualitative changes in both leptin-estradiol and IGF-I-estradiol inter-relationships could be involved in the development of reproductive aging in this species.

The functional inter-relationships between leptin and IGF-I are not to be excluded. At least, the influence of leptin on ovarian IGF-I receptor (ewe; Muñoz-Gutiérrez et al., 2005), the effect of IGF-I on ovarian leptin receptors (pig; Gregoraszczuk et al., 2007), and the ability of leptin to modify the steroidogenic effect of IGF-I (humans; Karamouti et al., 2008) and vice versa (human; Sirotkin et al., 2005) have been reported. The comparison of leptin and IGF-I effects on mink steroidogenesis suggest the antagonistic interrelationships between leptin and IGF-I in the control of ovarian estradiol and aging, but the existence and mechanisms of such inter-relationships in this species require further studies.

Taken together, our observations demonstrate the involvement of both leptin and IGF-I in the control of mink ovarian steroid hormone release. Furthermore, the findings suggest that reproductive aging can be due to (a) reduction in basal progesterone release and (b) alterations in response of estradiol (but not of progesterone) to leptin and IGF-I. Further studies are needed to help elucidate the processes of reproductive aging in minks (an important farm animal and a model of reproductive biology) and to contribute to the solution to reproductive problems in other animals and humans.

## MATERIALS AND METHODS

The present study was performed on female American minks of standard variation kept individually in metal cages at the Liesek farm (Čimhova, Slovakia) in standard conditions and feed ration (Mertin et al., 1994; Süvegová et al., 2000). All the animals were in a good clinical and metabolic state with good fur quality and reproductive active before sample collection. In December, after completion of the reproductive cycle and pup rearing, during pelting, the animals were anesthetized by intramuscular injection of xylazin (Bioveta, IvanovicenaHane, Czech Republic, 1 mg/animal) and then euthanized using cardiac injections of T61 (Intervet International BV, Boxmeer, Netherlands, 500 mg/animal) in accordance with Slovak and EU regulations in animal welfare. Two groups of animals were compared – young (yearlings) and old (3-5 years of age).

Immediately after pelting, the collected ovaries were transported to the laboratory in sterile physiological solution (0.9% NaCl); two hours after collection, the ovaries were dissected into quarters, washed three times in sterile physiological solution and cultured 48 h in Dulbecco's modified Eagle's medium (DMEM)/F-12 1:1+10% calf fetal serum+1% antibiotic-antimycotic solution (all from Sigma, St. Louis, MO, USA) in Falcon 24-well plates (Becton Dickinson, Lincoln Park, NJ, USA), 2 ml medium per well. In addition, the samples were treated with 0, 1, 10 or 100 ng/ml of leptin and IGF-I (biological grade; Sigma). These concentrations correspond the levels of hormones expected in the mink blood (Sundqvist et al., 1989; Tauson, 2001) and the concentrations of IGF-I (Chrenek et al., 2010; Maruniakova et al., 2015) and leptin (Sirotkin and Grossmann, 2007; Maruniakova et al., 2015) affecting the secretory activity of cultured ovarian fragments in other species. The hormones were dissolved in culture medium immediately before the experiment. After two days of culture, the medium from the 24-well plates was aspirated and frozen at −18°C to await radioimmunoassay (RIA).

### Immunoassays

Concentrations of progesterone and estradiol were determined by RIA in 25-100 µl samples of incubation medium previously validated for use as a cell culture medium. We assayed progesterone using our own RIA systems (Kotwica and Skarzynski, 1993). The antiserum against progesterone has less than 0.001% cross-reactivity to cortisol, corticosterone, androstenediol, pregnenolone, estradiol and testosterone. The sensitivity of the assay was 0.12 ng/ml, the maximal intra- and inter-assay coefficients of variation were 13.1 and 8.0%, respectively. Estradiol was assayed using RIA kits from DSL (Webster, TX, USA) according to the instructions of the manufacturer. The antiserum used cross-reacted less than 0.01% with dehydroepiandrosterone, progesterone, cortisol, danazol, androsterone, testosterone, androstenedione, corticosterone, and cortisone. The sensitivity of RIA was 9.4 ng/ml, the intra- and inter-assay coefficients of variation were 9.4 and 19.0.

### Statistics

The data shown are the means of values obtained in two separate experiments performed on separate days with separate groups of ovaries, each obtained from 10-15 animals. Each experimental group was represented by six culture wells. Assays of hormonal content in the incubation medium were performed in duplicate. After RIA, the values of blank control (medium cultured without cell or exogenous hormones) were subtracted from the value determined in cell-conditioned serum-supplemented medium to exclude any non-specific background (less than 10% of total values). The rates of substance secretion were calculated per mg ovarian tissue/day. Significant differences between the experiments were evaluated using two-way ANOVA. When the effects of treatments were revealed, data from the experimental and control groups were compared with the Wilcoxon–Mann–Whitney multiple range test using the SigmaPlot/SigmaStat program, version 11.0 (Systat Software, GmbH, Erkhart, Germany). Differences from control at *P*<0.05 were considered significant.
